# Lower pain and higher muscular strength in immigrant women with vitamin D deficiency following vitamin D treatment

**DOI:** 10.1080/22423982.2017.1340547

**Published:** 2017-08-03

**Authors:** Marianne Englund, Jan Persson, Ingrid Bergström

**Affiliations:** ^a^ Department of surgery at Danderyds Hospital, Stockholm, Sweden; ^b^ Department of Anaesthesia and Intensive Care, Karolinska University Hospital Stockholm, Stockholm, Sweden; ^c^ Clinical Science, Intervention, and Technology, Karolinska Institutet, Stockholm, Sweden; ^d^ Department of Endocrinology, Metabolism, and Diabetes, Karolinska University Hospital, Stockholm, Sweden

**Keywords:** Vitamin D, Nordic region, immigrants, musculoskeletal pain, muscular strength

## Abstract

**Background**: Vitamin D deficiency is common among immigrants in the Nordic region. It may lead to osteomalacia with severe musculoskeletal pain. There are reports that vitamin D deficiency without osteomalacia may lead to pain but little is known of the effect of treatment.

**Objective**: To investigate whether a moderate dose of cholecalciferol and calcium improves strength and pain in a group of vitamin D deficient women.

**Design**: Twentyfive immigrant women with vitamin D deficiency diagnosed during pregnancy were treated postpartum with a daily dose of 1,600 IU cholecalciferol and 1,000 mg of calcium. They were examined at the start of treatment and again after 3 months of treatement and the results were statistically compared. Setting: Southern parts of Stockholm.

Main outcome measures: Serum 25-hydroyvitamin D (25(OH)D), serum-parathyroid hormone (PTH), pain measured by a visual analogue scale (VAS), musculoskeletal strength by performance on a chair stand test (seconds), and bone tenderness by pressure algometer (kilo-Pascal).

**Results**: Following the treatment, the 21 women attending had lowered cm in VAS, improved musculoskeletal strength, - and 25(OH)D levels were normalized.

**Conclusions**: A moderate dose of vitamin D normalized l vitamin D levels, improved muscular strength and reduced pain in this group of vitamin D deficient immigrant women.

## Introduction

In the Nordic region, 2 factors that contribute to the high prevalence of vitamin D deficiency among non-Western immigrants are considered to be darker skin tone and lower sun exposure owing to traditional behaviour and the use of clothing that covers much of the skin [1,2].

### Vitamin D and pain

Persistently low levels of vitamin D might lead to a mineralisation defect (osteomalacia) and a compensatory parathyroid hormone (PTH) increase (secondary hyperparathyroidism [2]. Severe symmetrical musculoskeletal pain in the lower back and lower extremities and muscular weakness are associated with osteomalacia [3]; bone tenderness is another sign of the condition [3]. In addition, it has been reported that vitamin D deficiency may itself cause muscle and bone pain [4]. In the past 40 years, there have been reports of patients with symmetrical musculoskeletal pain related to vitamin D deficiency but without evidence of osteomalacia [5]. This may represent a long-lasting pain that occurs before the development of osteomalacia [4]. The association between musculoskeletal pain and vitamin D deficiency has been documented in women living in Saudi Arabia [6], and in immigrant populations in Europe and North America [7–9]. Knutsen et al. found a high prevalence of vitamin D deficiency (58%) in a group of patients, both ethnic and non-ethnic, who reported musculoskeletal pain and headache. The prevalence of hypovitaminosis was higher among the non-natives (83 versus 35 percent) [10]. Only a few treatment studies have examined the effect of vitamin D treatment on musculoskeletal pain in vitamin D-deficient immigrant women; however, the results of these have been contradictory [6,7,9,11–13].

### Vitamin D and muscular function

Vitamin D receptors (VDRs) are present on skeletal muscle fibres, and genetic variations in VDRs have been shown to correlate with muscle strength [14]. A recently published study found an association between the vitamin D status of young Somali immigrant women in Sweden and their muscle strength [15]. In the elderly, the association of vitamin D and calcium treatment with improved muscular function is well established [16]. A meta-analysis found substantially higher proximal muscle strength following vitamin D treatment in vitamin D-deficient individuals [17]. However, one randomised double-blind placebo-controlled trial from Norway did not find any effect of vitamin D treatment on muscular function in vitamin D-deficient individuals [18].

The primary aim of this study was to determine whether a treatment consisting of a moderate dose of vitamin D and calcium over 3 months is associated with improvement in musculoskeletal pain and muscular strength in vitamin D-deficient immigrant women living in Stockholm, Sweden. A secondary aim was to investigate whether the treatment was enough to normalise vitamin D levels.

## Material and method

### Patients

Twenty immigrant women with 25(OH)D values < 25 nmol/L and 5 immigrant women with 25(OH)D values < 38 nmol/L from an earlier observational study of vitamin D levels and pain in pregnant immigrant women living in Sweden [2] started postpartum treatment with vitamin D and calcium. The women originated from the African continent, Bangladesh, the Middle East, India, Pakistan and South America. At the time of the study the women had residence in Stockholm, Sweden (58°N). The time they had been living in Sweden varied in the range of 7 and 12 years. They had not been treated with vitamin D before they were recruited to the earlier observational study or during that study. As they were identified with low vitamin D levels in the observational study they were offered treatment with vitamin D and calcium and follow-up according to the earlier ethical approval (2007/1224–31). They were prescribed combination pills with a dose of 1,600 IU cholecalciferol, in this manuscript referred to as vitamin D, and 1,000 mg calcium. The pills were taken on a daily basis. Most of the women were of light to medium skin tone. They dressed in a European style, but a majority avoided exposing both arms and legs to sunlight during the summer months.

### Statement of human rights

The follow-up was approved by the local ethics committee (No. 2010/632–32). Each of the women received oral and written information, and each signed an informed consent form.

### Blood sampling

Non-fasting blood samples were drawn and analysed at baseline and again after 3 months. The samples were taken consecutively upon recruitment regardless of the season. The following biomarkers were analysed in heparinised plasma using DXC800 (Beckman Coulter, Brea, CA, USA): calcium, phosphate, albumin, alkaline phosphatase (ALP) and creatinine. All these assays had a total coefficient of variation (CV) of less than 3%. Free (ionised) calcium was measured using an ABL 800 (Radiometer, A/S Copenhagen, Denmark). Participants’ 25(OH)D in serum was determined using a competitive chemiluminescence assay (CLIA), LIAISON (DiaSorin Inc., Saluggia, Italy), with a total CV of less than 8% for samples of a concentration between 10 and 128 ng/mL (25–310 nmol/L). Intact PTH in plasma (reference values: 10-65 nmol/l) was analysed using a Modular Analytic E System (Roche Diagnostics GMBA, Manheim, Germany), with a total CV of less than 3%.

### Pain evaluation

Musculoskeletal pain and bone tenderness were evaluated by the clinical doctor at start of treatment and at a follow-up visit after 3 months.

Musculoskeletal pain was scored as the maximum pain during the past week on a visual analogue scale (VAS) 10/10 [19]. Any general symmetric pain in the back, legs or arms was considered musculoskeletal pain.

Bone tenderness was evaluated with a pressure algometer applied to the middle of the sternum (Somedic Production AB, Sollentuna, Sweden) [20,21]. When the subject experienced the pressure as painful, she was instructed to press a button, thereby locking the pressure gauge at the pain-threshold value. The tests were performed by 1 examiner per patient, and 3 different examiners were involved.

### Muscular performance

Muscular performance was evaluated at baseline and again after 3 months with a leg-muscle performance test. A timed stand test was used during which the time (in seconds) needed to stand up 10 times from a standard chair was recorded [22].

### Statistical analysis

Statistical analysis was performed with IBM® SPSS version 20.0 (SPSS, Chicago, IL, USA). Visual inspection was performed in order to check for normal distribution. We observed high levels of divergence in all variables except VAS and the timed stand test. To make statistical comparisons, *t*-tests and the non-parametric exact Wilcox test were used. In addition, non-parametric regression and analyses of correlations of the different test variables were performed. The results are presented as means, standard deviations (SD), and 2-sided p-values. Results of p<.05 were considered significant.

## Results

For baseline characteristics, see Table 1. During the 3 treatment months, 3 subjects became pregnant. One withdrew from the study before the 3-month evaluation. As pregnancy can interfere in the deterioration or improvement of perceived pain or muscular performance, the results of these examinations were excluded. There was no significant difference in age, weight, height, body mass index (BMI), 25(OH)D levels, or PTH levels between the 4 women excluded and the 21 women who completed the study. (Table 1).

**Table 1.** Descriptive statistics (mean, SD, minimum, and maximum) at baseline investigations for the treatment study group. Rated pain is an assessment of the maximum pain the past week on VAS 10/10. Bone tenderness measured with an algometer pressed against the sternum.

	The whole treatment study group				The attending		The non-attending	
	n=25				n=21 (84%)		n=4 (16%)	
	Mean	Min.	Max.	SD	Mean	SD	Mean	SD
Age (years)	32.0	21.5	40.5	4.7	31.7	5.0	33.7	2.4
Height (cm)	159.2	145	172	6.3	159.0	6.2	160.3	7.8
Weight (kilogram)	70.6	52	90	11.7	70.4	12.3	71.8	8.4
BMI	27.9	19	36	4.6	27.9	4.9	28.0	2.7
25(OH)D (nmol/L)	19.8	10	38	9.2	20.0	8.9	19.0	12.3
PTH (nmol/L)	80.5	22	175	43.2	80.4	46.5	81.3	22.7
Rated pain in VAS	5.7	2	10	2.1	5.7	2.1	no value	no value
Chair stand test* (s)	23.1	12	37	5.8	23.4	5.8	20.5	7.8
Bone tenderness (kPa)	584	210	876	186.4	567.7	184.1	721	202.2

VAS, visual analogue scale; PTH, parathyroid hormone; 25(OH)D, 25-hydroxyvitamin D.

* Time taken to stand up from sitting position 10 times.

There was a significant increase in vitamin D levels after 3 months of treatment with vitamin D and calcium. The PTH levels were significantly lower. ALP was normal and did not change (not shown in table). The immigrant women demonstrated significantly higher muscular strength, and the pain measured on VAS was lower. Their perceived tenderness of the bone did not change from baseline and after 3 months. Not all the women in the study underwent all the tests. (Table 2).

**Table 2.** The results (mean, maximum, minimum and p-values) of the investigations following 3 months of treatment compared with the results at baseline.

	Baseline				Following 3 mos of treatment				Difference of	Significance
	Mean	Min.	Max.	*n*	Mean	Min.	Max.	*n*	the means	P-value
25(OH)D (nmol/L)	20.0	10	38	21	61.3	36	119	19	41.3	<0.001
PTH (nmol/L)	80.4	22	175	21	47.3	18	110	21	−33.1	0.004
Rated pain in VAS (cm)	5.7	2	10	19	3.2	0	7	18	−2.5	0.005
Chair stand test (s)	23.4	12	37	21	19.7	11	29	20	−3.7	<0.001
Bone tenderness (kPa)	567.7	210	876	17	542.5	239	882	0	−25.2	0.470

VAS, visual analogue scale; PTH, parathyroid hormone; 25(OH)D, 25-hydroxyvitamin D.

Regression analysis of the change in 25(OH)D and PTH during the treatment period did not predict the outcome of the different tests performed at follow-up. Neither did we find any correlation between the change in 25(OH)D or PTH and the results of investigation at the follow-up.

## Discussion

After we treated immigrant women who were vitamin D deficient with a moderate dose of vitamin D and calcium for 3 months, their vitamin D levels were normalised, pain was decreased and muscular strength was improved. These women, who had lived in Sweden for over 7 years, had never been treated with vitamin D and were vitamin D deficient, with a mean value of 20 nmol/L at the start of treatment. When treatment started, Sweden had no clinical consensus of doses of vitamin D for vitamin D deficiency. We treated these women with 1,600 IU of cholecalciferol and 1,000 mg of calcium. The cholecalciferol dose was double the recommended dose for patients with osteoporosis who have sufficient vitamin D levels. With this moderate dose of cholecalciferol, the treatment protocol ensured that the patients would not have possible prolonged side-effects (e.g. increased mortality) [23]. If this cholecalciferol dosage was insufficient, then we could increase the dose. In addition, the calcium treatment (i.e. 1000 mg) was chosen to secure the daily needed calcium intake, which is part the recommended treatment for osteoporosis.

Because it would be unethical not to treat vitamin D-deficient patients, we did not perform a randomisation study. Therefore, we performed an observational report of a clinical treatment procedure and its clinical effects. Thus, a limitation is the lack of a control group. Another limitation is how we determined 25(OH)D – i.e. the CLIA. This method has been shown to overestimate the number of samples with low 25(OH)D and with a high CV compared with other methods [24]. The strength in our clinical observational study is that the serum level of vitamin D was measured before and after the study.

The findings of an earlier observational study on the same study group indicated a correlation between musculoskeletal pain and vitamin D levels [2]. In a clinical meeting, we evaluated pain as well as muscular performance in vitamin D-deficient patients and during treatment. We found that the women’s perceived muscular skeletal pain measured on a VAS scale decreased, and also found an improvement in muscular strength. This supports the findings of Torrenté et al. [9], who also saw reversal of pain following 3 months of treatment in a majority of female asylum seekers, and corroborates the findings of others as well [6,11]. However, Helliwell et al. [7] saw no treatment effect of vitamin D on pain; this was possibly due to low compliance, as the effects on biochemical markers were also low in that study.

The increase in muscular strength observed in our study group differs from the findings by Knutsen et al. who, in their randomised study, saw no improvement in muscular strength [18]. It has been suggested that bone tenderness can also be used to diagnose osteomalacia [9], and that pressure sensitivity of the sternum and the edge of the tibia, radius or ulna has diagnostic value [20,21]. That bone tenderness in the group of immigrant women did not change might be a sign that the women did not suffer from osteomalacia. There was a significant difference in the PTH levels before and after vitamin D treatment. However, the PTH values were only slightly above normal range at the baseline and the ALP was normal. Presumably, this might be another sign that osteomalacia had not yet occurred in this group of immigrant women, in spite of the persistent low vitamin D levels. According to Bhan et al., vitamin D deficiency in the early stages, along with increased serum levels of PTH and ALP, is associated with increased bone turnover, but without mineralisation defect or cortical bone loss [3]. Later, at the hypovitaminosis D osteopathy stage III (HVO III), there is a complete cessation of mineralisation consistent with the traditional descriptions of osteomalacia. However, osteomalacia can only be diagnosed by a bone biopsy [3]. A single causative mechanism has not been identified that explains the pain associated with vitamin D deficiency in the absence of osteomalacia. We agree with Kragstrup that treatment should be considered in patients with chronic pain, as vitamin D treatment is relative safe and testing is cheap [25]. Moreover, we see a need for more randomised controlled treatment studies on the effect of vitamin D on muscular strength and on bone and muscle pain.

## Conclusion

The results indicate that vitamin D treatment can lead to lower levels of pain and improve muscular function. A moderate dose of cholecalciferol for 3 months increased vitamin D levels to normal values in immigrant women with vitamin D deficiency. The prevalence of vitamin D deficiency among immigrants living in Sweden is high. Vitamin D testing and treatment should be considered in immigrant women to avoid the risk of developing the severe condition of osteomalacia.

## Key points

- Deficiency of vitamin D is a common condition and is associated with musculoskeletal pain and weakness.
- Following a short treatment period with vitamin D and calcium a group of young, healthy immigrant women had their vitamin D levels normalised.
- Higher muscular strength (chair stand test) and lower pain were registered on the VAS.
- Clinicians should be aware of the need for testing and treating at-risk groups, including immigrants.
